# Genetic tracing uncovers the importance of epithelial-to-mesenchymal transition in small cell lung cancer chemotherapy resistance but not metastasis

**DOI:** 10.1038/s41421-024-00687-8

**Published:** 2024-06-04

**Authors:** Yuting Chen, Chenchen Guo, Xinlei Cai, Liang Hu, Xinyuan Tong, Yun Xue, Qiqi Zhao, Tengfei Zhang, Yuan Chen, Yongting Fang, Yayi He, Yan Li, Bin Zhou, Hongbin Ji

**Affiliations:** 1 School of Life Science and Technology, ShanghaiTech University, Shanghai, China; 2 Key Laboratory of Multi-Cell Systems, Shanghai Institute of Biochemistry and Cell Biology, Center for Excellence in Molecular Cell Science, Chinese Academy of Sciences, Shanghai, China; 3 School of Life Science, Hangzhou Institute for Advanced Study, University of Chinese Academy of Sciences, Hangzhou, Zhejiang China; 4 University of Chinese Academy of Sciences, Beijing, China; 5 Department of Medical Oncology, Shanghai Pulmonary Hospital, School of Medicine, Tongji University, Shanghai, China; 6 Shandong Laboratory of Yantai Drug Discovery, Bohai Rim Advanced Research Institute for Drug Discovery, Yantai, Shandong China; 7 State Key Laboratory of Drug Research, Shanghai Institute of Materia Medica, Chinese Academy of Sciences, Shanghai, China

**Keywords:** Chemotherapy, Epithelial-mesenchymal transition

Dear Editor,

Small cell lung cancer (SCLC), a high-grade neuroendocrine carcinoma, is frequently associated with poor prognosis due to high metastases^1^. Standard chemotherapy improves patient survival by several months, but chemotherapy resistance emerges rapidly^2^. Epithelial-to-mesenchymal transition (EMT) is important for cancer metastasis and drug resistance involving E-cadherin downregulation and Vimentin (VIM) upregulation^3–5^. SCLC exhibits elevated VIM expression and other EMT-related transcription factors TWIST1 and ZEB1^6^. Targeting EMT potentially reverses SCLC chemotherapy resistance^7^. However, whether EMT occurs in vivo and its functional significance during tumor growth, metastasis, and drug resistance remains largely elusive.

**Fig. 1:**
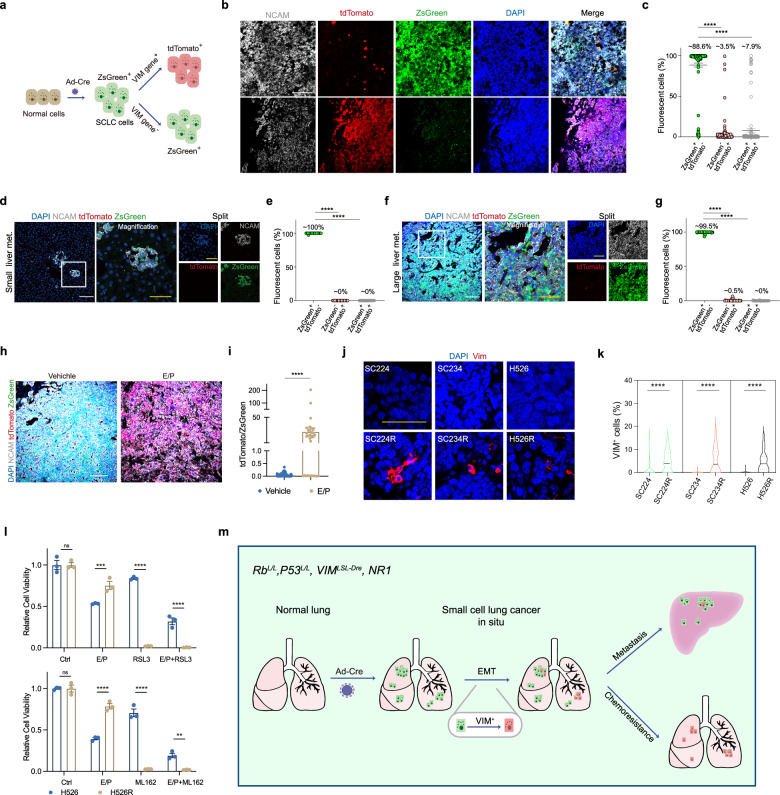
Genetic tracing of EMT in SCLC metastasis and chemotherapy resistance. **a** Schematic diagram showing the different fluorescence reporters. **b**–**g** Immunostaining of NCAM, ZsGreen and tdTomato on primary tumors (**b**) or small metastases (**d**) or large metastases (**f**). met metastases. Quantification of the percentage of ZsGreen^+^tdTomato^–^, ZsGreen^+^tdTomato^+^, ZsGreen^–^tdTomato^+^ cells in NCAM^+^ SCLC cells in primary tumors (139 fields/4 mice) (**c**) or small metastases (26 fields/4 mice) (**e**) or large metastases (44 fields/4 mice) (**g**). **h** Immunostaining of NCAM, ZsGreen and tdTomato on lungs from mice treated with vehicle and E/P. **i** Quantification of the ratio of ZsGreen^–^tdTomato^+^ to ZsGreen^+^tdTomato^–^ in vehicle (71 fields/4 mice) and E/P groups (30 fields/5 mice). **j** Immunostaining of VIM on PDX/CDX models. **k** Quantification of the percentages of VIM^+^ cells in the SC224 (27 fields/3 mice), SC224R (29 fields/3 mice), SC234 (33 fields/3 mice), SC234R (31 fields/3 mice), H526 (29 fields/3 mice) and H526R (31 fields/3 mice) models. **l** Relative viability of H526 and H526R cells following 72-h treatment of E/P, ML162, RSL3 alone or in combination (*n* = 3 technical replicates). **m** Schematic diagram indicating the importance of EMT in SCLC chemotherapy resistance but not metastasis using *Rb1*^*L/L*^;*Trp53*^*L/L*^;*VIM-Tracer* model. White scale bars = 100 µm, yellow scale bars = 50 µm. Data represent means ± SEM. One-way ANOVA with multiple comparisons test. Mann–Whitney test. Two-way ANOVA with multiple comparisons test. **P < 0.01, ***P < 0.001, ****P < 0.0001.

Based on frequent concurrent genetic inactivation of *RB1* and *TP53* in human SCLC, the *Rb1*^*L/L*^;*Trp53*^*L/L*^ autochthonous SCLC mouse model is developed to recapitulate SCLC development and liver metastasis^8^. We have recently developed a *Vimentin*^*LSL-Dre*^ and *nested reporter 1* (*NR1*) mice, referred to as *VIM-Tracer*, for tracing EMT in breast cancer mouse model^9^. In this system, Dre recombinase activity is driven by *Vimentin* promoter after initial inducible Cre-loxP recombination. The switch from ZsGreen to tdTomato indicates the VIM expression and thus permanently records VIM-mediated EMT activity^9^. We here generated the *Rb1*^*L/L*^;*Trp53*^*L/L*^;*VIM-Tracer* mice to trace EMT in SCLC mouse model (Fig. 1a; Supplementary Fig. S1a). These mice develop SCLC positive for ZsGreen after Adeno-Cre (Ad-Cre) treatment (Fig. 1a; Supplementary Fig. S1b)^8^. When EMT occurs, the *Vimentin* gene and its driven Dre recombinase are expressed, resulting in Dre-rox recombination to switch the reporter from ZsGreen to tdTomato (Fig. 1a)^9^. Thus, the detection of tdTomato positivity enables us to trace VIM-associated EMT occurrence in vivo. This system permits a seamless recording of EMT, even transiently occurring during tumor malignant progression^9^.

In *Rb1*^*L/L*^;*Trp53*^*L/L*^ model, SCLC initially develops ~24 weeks post-Ad-Cre treatment and metastasizes into the liver ~40 weeks afterwards. To trace the EMT process, we collected mouse lungs and livers at 40 weeks post-Ad-Cre treatment and found that all tumors were ZsGreen^+^ in whole-mount fluorescence imaging (Supplementary Fig. S1b, c). Genotyping analyses of primary tumors confirmed the deletion of a *Stop* codon in the *Vimentin*^*LSL-Dre*^ allele (Supplementary Fig. S1d). Immunofluorescence staining showed that tdTomato^+^ cells expressed VIM (Supplementary Fig. S1e). Both ZsGreen^+^tdTomato^–^ and ZsGreen^–^tdTomato^+^ tumor cells highly expressed the neuroendocrine marker neural cell adhesion molecule (NCAM)^10^ (Fig. 1b; Supplementary Fig. S1f). Both the Notch pathway and YAP are known to be important for the SCLC fate switch between neuroendocrine and non-neuroendocrine^11,12^. Immunostaining data showed that the ZsGreen^–^tdTomato^+^ cells did not have notable HES1 or YAP expression (Supplementary Fig. S1g–j), indicating that these ZsGreen^–^tdTomato^+^ cells likely belong to the neuroendocrine subtype without obvious HES1 or YAP expression.

Gross inspection showed almost no tdTomato^+^ signals in primary tumors (Supplementary Fig. S1c). This was further confirmed by immunofluorescence analyses showing that most NCAM^+^ SCLC cells (~88.6%) were ZsGreen^+^tdTomato^–^ (Fig. 1b, c). Despite the low percentages of tdTomato^+^ cells, a few tumors uniformly displayed a tdTomato^+^ pattern (Fig. 1b, c). These observations demonstrated the existence of EMT in primary SCLC, albeit at a low incidence. Consistent with our previous study^9^, ~7.9% of SCLC cells were double positive for ZsGreen and tdTomato, indicative of a transitioning state with initial tdTomato expression yet non-degraded ZsGreen (Fig. 1b, c).

We further found that most liver metastases were ZsGreen^+^tdTomato^–^ (Supplementary Fig. S2a). Immunofluorescence analyses showed that small liver metastases (diameter < 100 µm) were uniformly ZsGreen^+^tdTomato^–^ (Fig. 1d, e). Large liver metastases (diameter > 500 µm) were found to have a sparse distribution of ZsGreen^–^tdTomato^+^ cells among the vast majority of ZsGreen^+^tdTomato^–^ cells (Fig. 1f, g). No metastases were uniformly ZsGreen^–^tdTomato^+^. These data indicate that tumor cells undergoing EMT have no detectable advantage in SCLC metastases. Further analyses of the TCGA dataset and clustered 81 SCLC patients based on VIM expression^13^ showed that VIM expression was not associated with patient prognosis (Supplementary Fig. S2b). Moreover, we found no significant correlation between SCLC metastasis and VIM expression or EMT score in these patients (Supplementary Fig. S2c), suggesting that VIM might not be a predominant factor in regulating SCLC metastasis.

Emerging studies suggest a link between EMT and chemotherapy resistance^3,4,7^. Etoposide and cisplatin (E/P) have been established as the first-line treatment for SCLC for past decades^2^. At 38 weeks post-Ad-Cre treatment, we treated the *Rb1*^*L/L*^;*Trp53*^*L/L*^;*VIM-Tracer* mice with E/P for 4 cycles following standard protocol (Supplementary Fig. S3a). Interestingly, we observed significant tumor regression in response to chemotherapy (Supplementary Fig. S3b–d). The ratio of ZsGreen^–^tdTomato^+^ to ZsGreen^+^tdTomato^–^ greatly increased following E/P treatment, and most residual SCLC were ZsGreen^–^tdTomato^+^ (Fig. 1h, i). These ZsGreen^–^tdTomato^+^ tumor cells showed decreased cleaved caspase-3 (CC3) (Supplementary Fig. S3e, f). Moreover, we did not observe significant differences in Ki-67 (proliferation marker) or P21 (cell cycle marker) signal between ZsGreen^+^tdTomato^–^ and ZsGreen^–^tdTomato^+^ cells before and after chemotherapy (Supplementary Fig. S3g–j).

We further analyzed VIM expression in chemotherapy-resistant SCLC patient-derived xenograft (PDX) and cell-derived xenograft (CDX) models established previously^14^. After up to 18 months of intermittent E/P treatments, these PDX tumors (SC224, SC234) and CDX tumors (H526) became resistant to chemotherapy, respectively named as SC224R, SC234R and H526R^14^. Immunofluorescence analyses showed increased VIM expression in all these resistant tumors (Fig. 1j, k), implicating a potential role of EMT in SCLC chemotherapy resistance.

We next asked whether targeting EMT could overcome SCLC chemotherapy resistance. Inhibition of GPX4 with ML162 or RSL3 could induce ferroptosis in therapy-resistant cancer cells at the mesenchymal state^15^. To evaluate the inhibitory effects of GPX4 targeting, we took advantage of H526R (chemotherapy-resistant) and H526 (chemotherapy-sensitive) cell lines (Fig. 1l)^14^ for assessing the IC50 values of RSL3 and ML162 treatments (Supplementary Fig. S3k, l). We chose the dose of RSL3 at 0.025 µM and ML162 at 0.075 µM, which preferentially suppressed the growth of H526R but not H526 cells (Fig. 1l). We found that either RSL3 or ML162 alone or in combination with E/P significantly inhibited H526R cell growth (Fig. 1l), suggesting that EMT targeting might serve as a therapeutic vulnerability for chemotherapy-resistant SCLC.

Utilizing the dual homologous recombination system, we find that EMT does exist in SCLC malignant progression. However, EMT seems not associated with SCLC metastasis but very likely contributes to SCLC chemotherapy resistance (Fig. 1m). This aligns with previous studies in breast and pancreatic cancers, underscoring the importance of EMT specifically in chemotherapy resistance rather than metastasis^3,4^.

Using the *Rb1*^*L/L*^;*Trp53*^*L/L*^;*VIM-Tracer* model, we found no tumor or metastasis lesion showing 100% tdTomato^+^ cells. Instead, we observed tdTomato^+^ cells dispersedly distributed at a low percentage in both primary and metastatic tumors. Considering the transient or stochastic expression of VIM during malignant progression, our lineage tracing system might overestimate the number of cells and lineages that have committed to the EMT fate. This indicates that mesenchymal transition does not confer cancer cells advantages in survival or expansion during SCLC metastasis, in accordance with previous studies^3,4,9^. Moreover, we found no association between VIM expression and human SCLC metastasis. Of note, most TCGA data used in this study were from surgically resected SCLC and EMT might not be present. Future studies are necessary to validate the clinical relevance of our findings at extensive-stage SCLC. Previous studies have uncovered important mechanisms involving SCLC metastasis, potentially via NFIB, Cullin5, etc.^1^. It remains possible that other molecular mechanisms instead of EMT play important roles in promoting SCLC metastasis.

Moreover, we uncovered the potential role of EMT in SCLC chemotherapy resistance using the SCLC mouse model, PDX and CDX models. In the SCLC mouse model, E/P treatment dramatically reduced ZsGreen^+^ tumors, and most residual tumors were consistently tdTomato^+^, which might be present before drug administration. These tdTomato^+^ tumor cells are capable to survive chemotherapy due to apoptotic tolerance. Consistently, we found VIM upregulation in E/P-resistant human SCLC PDX and CDX models. These data provided the first in vivo genetic evidence connecting EMT and SCLC chemotherapy resistance. Indeed, these findings align with previous findings of high VIM expression after chemotherapy in SCLC CDX models^6^. Strategies to target the EMT program are emerging. Deletion of *Twist1* or *Snai1* sensitizes pancreatic cancer to gemcitabine treatment^4^, and miR-200 overexpression abrogates CTX resistance in breast cancer^3^. We found that ML162 and RSL3 treatments preferentially suppress the growth of chemotherapy-resistant SCLC in vitro. Further study is required to validate whether combined conventional chemotherapy and EMT-targeted therapy could effectively overcome SCLC drug resistance in vivo. We have not assessed whether apoptosis or ferroptosis is the major form of cell death in this setting. Given that the ZsGreen^–^tdTomato^+^ tumor cells showed decreased levels of CC3 after chemotherapy, it remains possible that apoptotic inducer treatment might represent a potential strategy to overcome SCLC resistance.

### Supplementary information

Supplementary information
