# Supplementary material for: Genetic tracing uncovers the importance of epithelial-to-mesenchymal transition in small cell lung cancer chemotherapy resistance but not metastasis
Source: Cell Discov. 2024 Jun 4;10:60. doi: 10.1038/s41421-024-00687-8 (PMC11150244; doi:10.1038/s41421-024-00687-8)
Supplement: Supplementary file 1 — Supplementary information [file 41421_2024_687_MOESM1_ESM.pdf]

Supplementary figure 1

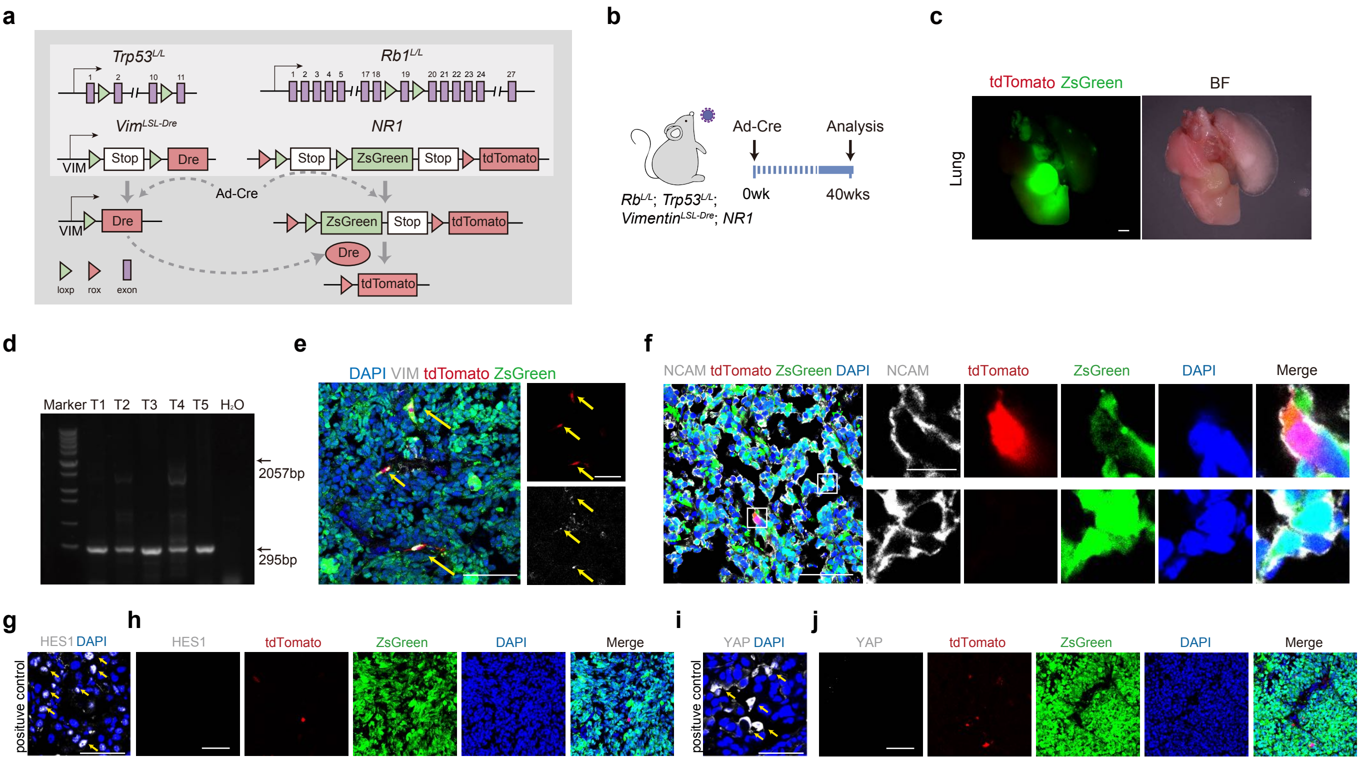

Supplementary figure 2

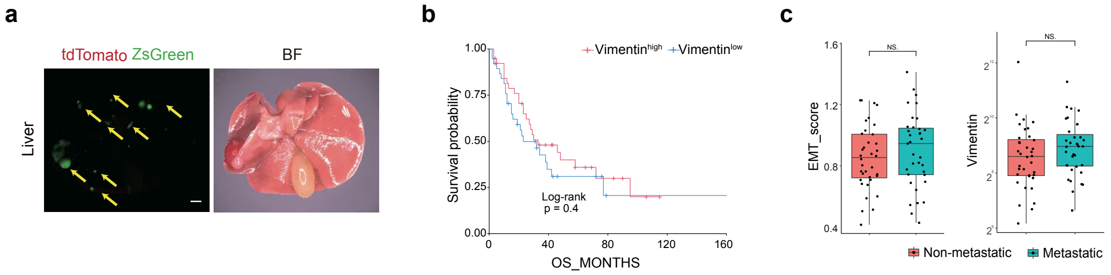

Supplementary figure 3

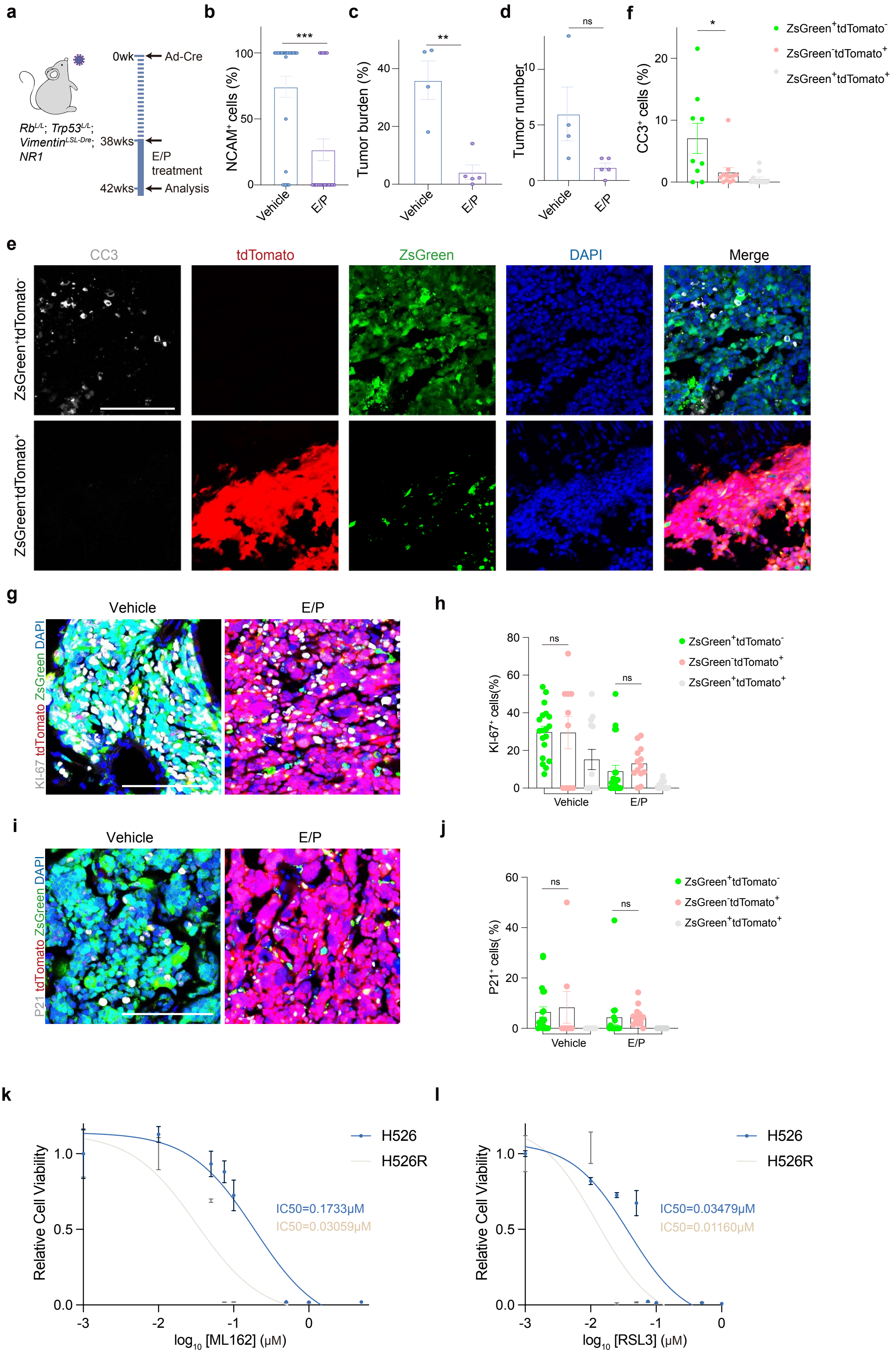

Supplementary Fig 1. Tracing EMT in primary tumors of *Rb1*$^{L/L}$;*Trp53*$^{L/L}$;*VIM-Tracer* mouse model

a Schematic illustration of *Rb1*$^{L/L}$;*Trp53*$^{L/L}$;*VIM-Tracer* model. **b** Schematic illustration of experimental details in *Rb1*$^{L/L}$;*Trp53*$^{L/L}$;*VIM-Tracer* mouse model. **c** Representative whole-mount fluorescence image of mouse lung post 40 weeks of Ad-Cre treatments. Scale bar = 2000 µm. BF: bright-field. **d** Genotyping of mouse lung tumors (T1-T5) from *Rb1*$^{L/L}$;*Trp53*$^{L/L}$;*VIM-Tracer* after Ad-Cre treatments. The band after stop codon deletion in the *Vimentin*$^{LSL\text{-}Dre}$ allele is 295bp and the band without deletion is 2057bp. **e** Immunostaining for Vimentin, ZsGreen and tdTomato on mouse lung sections. Scale bar = 50 µm. **f** Immunostaining for NCAM, ZsGreen and tdTomato on mouse lung sections. Scale bar = 50 µm and 10 µm in the low and high magnification field, respectively. **g, i** Immunostaining of HES1 (**g**) and YAP (**i**) on lung sections from *EGFR*^{L858R};*Trp53*$^{L/L}$ mice is used as a positive control. Scale bar = 50µm. **h** Immunostaining for HES1, ZsGreen and tdTomato on mouse lung sections. Scale bar = 50 µm. **j** Immunostaining for YAP, ZsGreen and tdTomato on mouse lung sections. Scale bar = 50 µm.

Supplementary Fig 2. Tracing EMT in liver metastases of *Rb1*$^{L/L}$;*Trp53*$^{L/L}$;*VIM-Tracer* mouse model

a Representative whole-mount fluorescence image of mouse liver post 40 weeks of Ad-Cre treatments. Scale bar = 2000 µm. BF: bright-field. **b** Kaplan-Meier curves for SCLC patient survival stratified by the median of Vimentin mRNA expression. *P* value

is established by the Mantel–Cox test. **c** Boxplots of Vimentin mRNA expression, EMT score in SCLC patients' tumors based on the metastasis status. EMT score is based on GSEA calculation. Unpaired t test.

Supplementary Fig 3. Tracing EMT in chemotherapy resistant SCLC in *Rb1^{L/L};Trp53^{L/L};VIM-Tracer*

a Schematic illustration of mouse experimental details. **b** Quantification of the percentages of SCLC cells (NCAM$^+$ cells) in vehicle (29 fields/4 mice) and E/P (30 fields/5 mice) groups. The p-values are calculated by fields. **c, d** Quantification of the tumor burden (**c**) and number (**d**) in vehicle (4 mice) and E/P (5 mice) groups. The p-values are calculated by mice. **e** Immunostaining for CC3 (cleaved caspase 3), ZsGreen and tdTomato on lung sections from mice treated with E/P. Scale bar = 100 µm**. f** Quantification of the percentages of CC3$^+$ cells in E/P groups (16 fields/5 mice). The p-values are calculated by fields. **g** Immunostaining for Ki-67, ZsGreen and tdTomato on lung sections in vehicle and E/P groups. Scale bar = 100 µm. **h** Quantification of the percentages of Ki-67$^+$ cells in ZsGreen$^+$tdTomato$^-$; ZsGreen$^-$tdTomato$^+$; ZsGreen$^+$tdTomato$^+$ in the vehicle (19 fields/4 mice) and E/P group (21 fields/5 mice). The p-values are calculated by fields. **i** Immunostaining for P21, ZsGreen and tdTomato on lung sections in vehicle and E/P groups. Scale bar = 100 µm. **j** Quantification of the percentages of P21$^+$ cells in ZsGreen$^+$tdTomato$^-$; ZsGreen$^-$tdTomato$^+$; ZsGreen$^+$tdTomato$^+$ in the vehicle (20 fields/4 mice) and E/P group (20 fields/5 mice). The p-values are calculated by fields. **k, l** IC50 analyses for ML162 and RSL3 in H526 and H526R cells (n = 3 technical replicates). Data represent means ± SEM. Unpaired t

test. One-way ANOVA with multiple comparisons test. Two-way ANOVA with multiple comparisons test. *$P < 0.05$, **$P < 0.01$ and ***$P < 0.001$. ns: not significant.

Supplementary materials and methods

Mouse models and chemotherapy treatments

All mice were housed at the Institutional Animal Care and Use Committee of the Institute of Biochemistry and Cell Biology, Chinese Academy of Sciences. All animal studies and procedures were conducted according to a protocol approved by the Animal Care and Use Committee at the Institute of Biochemistry and Cell Biology, Chinese Academy of Sciences. Conditional knockout *Rb1*$^{L/L}$ and *Trp53*$^{L/L}$ mice[1] were generously provided by Drs. Tyler Jacks, Ronald A. DePinho and Pierre Chambon. *Vimentin*$^{LSL\text{-}Dre}$ and *NR1* knock-in mouse lines were established as previously described[2]. To establish the system for trace EMT in SCLC mouse model, we crossed *Rb1*$^{L/L}$;*Trp53*$^{L/L}$ mice with *EMTgene*$^{LSL\text{-}Dre}$ (*Vimentin*$^{LSL\text{-}Dre}$); *NR1* mice. Both male and female mice at 6-8 weeks old were treated with adenovirus carrying Cre recombinase (Ad-Cre, 2x10^6 pfu) as previously described[3]. *Rb1*$^{L/L}$;*Trp53*$^{L/L}$;*VIM-Tracer* mice were given E/P treatment as previously described for 4 cycles before detailed pathological analyses [4]. For each cycle within a week time window, the etoposide (VP16, 10 mg/kg/d) was given at day 1-3 and cisplatin (DDP, 6 mg/kg/d) was given at day 1 as previously described[4].

The chemotherapy-sensitive and -resistant SCLC PDX and CDX mouse models were established as previously described[4]. All SC224R, SC234R and H526R showed strong resistance to E/P treatments[4].

Whole-mount fluorescence microscopy

Mice were anesthetized with chloral hydrate, and the blood was flushed out through right ventricle perfusion using PBS. Mouse lungs were dissected and placed on agar gel to obtain whole-mount bright-field and fluorescence images using a Zeiss stereoscope (AxioZoom V16).

Immunofluorescence of lung, liver and tumor sections

Mouse lungs, livers or tumors were dissected and fixed with 4% PFA and dehydrated in 30% sucrose overnight at 4°C and embedded in OCT (Thermo). Cryosections were about 10μm thickness and stored at −20 °C until use. The dried sections were washed with PBS and blocked with 3% normal goat serum in PBST (0.1% Triton X-100 in PBS). Sections were incubated with primary antibodies at 4°C overnight. After washing three times in PBS, sections were incubated with Alexa-conjugated secondary antibodies the next day (Cell Signaling Technology) for 40 minutes at room temperature. Followed by washing three times with PBS, nuclei were counterstained with DAPI. The following is primary antibodies: NCAM (Abclonal; A0393; 1:100), Vimentin (CST; 5741; 1:500). HES1 (CST; #11988; 1:100). YAP (Beyotime; AF5339, 1:100). Cleaved Caspase 3 (CST; #9661, 1:500), P21(Abcam; ab188224; 1:100), Ki-67(CST; #9129S; 1:100). For HES1, YAP, P21 and Ki-67 staining, horseradish peroxidase conjugated secondary antibody (Vector lab., MP-7401-50) were used in combination with Cyanine 5 Amplification Reagent (Akoya Biosciences, NEL745001KT, 1:1000). Pictures were taken by Leica TCS SP8 WLL or Leica TCS SP8 STED.

Genotyping

Mouse genomic DNA was extracted as previously described[3]. Following primers were used for genotyping the deletion of stop codon in the *Vimentin*$^{LSL\text{-}Dre}$ allele: forward, TTGTCCAGTCCTCTGCCACTCTTG; reverse, TACTCCTTGCCGATGTTCCTCAGG. The wildtype band is 2057bp and the band after the deletion of stop codon is 295bp.

Statistical analysis

Gene expression data of SCLC cancer patients were obtained from the cBioPortal (https://www.cbioportal.org/) after TPM normalization. We divided into two groups for the clinical data of SCLC patients by the median expression of vimentin and performed the survival analysis by “survival” and “survminer”, which are R packages for survival analysis. GSVA is an R package for calculate biological function score based on a single sample[5]. SCLC patients were divided into two groups based on lymphatic metastasis. We calculated the EMT score of SCLC samples by GSVA based on EMT signature that was from Li Wang’s study[6].

In vitro cell growth assay

Cisplatin (P4394, Sigma) were dissolved in saline. Etoposide (E1383, Sigma), RSL3 (HY-100218A, MCE) and ML162 (HY-100002, MCE) were dissolved DMSO. A total of 5000 H526 and H526R cells were dissociated in 100ul of RPMI medium containing 8% FBS and then seeded into a 96-well suspension plate. The next day, another 100ul of 8% FBS in RPMI medium with vehicle or inhibitors was added. Cell viability was measured using CellTiter-Glo 3D (DD1101-02, Promega) at 72h after treatment. For IC50 analysis,

ML162 was incubated at various concentrations ranging from 0.001 to 5µM, and RSL3 was tested across a concentration span of 0.001 to 1µM.

References

1 Meuwissen, R. *et al. Cancer Cell* **4**, 181-189 (2003).

2 Li, Y. *et al. Dev Cell* **54**, 593-607 e595 (2020).

3 Ji, H. *et al. Nature* **448**, 807-810 (2007).

4 Guo, C. *et al. Nat Cancer* **3**, 614-628 (2022).

5 Hanzelmann, S., Castelo, R. & Guinney, J. *BMC Bioinformatics* **14**, 7 (2013).

6 Wang, L. *et al. Nat Commun* **9**, 3503 (2018).
